# Measuring Engagement in Robot-Assisted Therapy for Autistic Children

**DOI:** 10.3390/bs13080618

**Published:** 2023-07-25

**Authors:** Abeer Al-Nafjan, Noura Alhakbani, Amal Alabdulkareem

**Affiliations:** 1 Computer Science Department, College of Computer and Information Sciences, Imam Mohammad Ibn Saud Islamic University (IMSIU), Riyadh 11432, Saudi Arabia; 2 Information Technology Department, College of Computer and Information Sciences, King Saud University, Riyadh 11543, Saudi Arabia; nhakbani@ksu.edu.sa (N.A.); 438203344@student.ksu.edu.sa (A.A.)

**Keywords:** robot, autism, therapy, engagement detection, facial emotion recognition, convolutional neural network

## Abstract

Children with autism face a range of challenges when it comes to verbal and nonverbal communication. It is essential that children participate in a variety of social, educational, and therapeutic activities to acquire knowledge that is essential for cognitive and social development. Recent studies have shown that children with autism may be interested in playing with an interactive robot. The robot can engage these children in ways that demonstrate and train essential aspects of human interaction, guiding them in therapeutic sessions to practice more complex forms of interaction found in social human-to-human interactions. This study sets out to investigate Robot-Assisted Autism Therapy (RAAT) and the use of artificial intelligence (AI) approaches for measuring the engagement of children during therapy sessions. The study population consisted of five native Arabic-speaking autistic children aged between 4 and 11 years old. The child–robot interaction was recorded by the robot camera and later used for analysis to detect engagement. The results show that the proposed system offers some accuracy in measuring the engagement of children with ASD. Our findings revealed that robot-assisted therapy is a promising field of application for intelligent social robots, especially to support autistic children in achieving their therapeutic and educational objectives.

## 1. Introduction

Autism Spectrum Disorders (ASDs) are a group of neurodevelopmental disorders that are characterized by persistent deficits in social communication and interaction, as well as restricted, repetitive patterns of behavior, interests, or activities, according to the Diagnostic and Statistical Manual of Mental Disorders [1]. ASD can manifest in a variety of ways, and symptoms can range from mild to severe. However, one common and significant challenge for children with ASD is social interaction and communication. They may struggle to initiate and maintain conversations, understand nonverbal cues, and engage in reciprocal play with peers. In addition, they may engage in repetitive behaviors or routines, which can interfere with their ability to learn and participate in social situations [2].

There is a strong tendency for traditional intervention approaches to require intensive support under the direct supervision of highly trained professionals. Many autistic individuals do not have access to professional care and amenities due to high intervention costs and/or a shortage of qualified therapists. Several interventions for children with ASD have been developed with the goals of improving cognitive ability and daily living skills, increasing their ability to interact and engage in the community, and trying to reduce symptoms. During therapy sessions, for example, assistive technologies have been used. This intervention is driven by the societal need for technological innovations that can support and improve current therapies for the increasing number of children with autism [3].

Today, a variety of assistive technology applications have been developed to assist in the treatment of autism [4], such as computer-assisted learning, virtual reality, telehealth, and robotics. For instance, Frolli et al. [5] investigated the use of virtual reality (VR) as a tool for improving social skills in individuals with ASD. The study compared emotional training using VR to traditional emotional training with a therapist and found that VR interventions can be effective in enhancing the acquisition of social skills, particularly for the use of primary and secondary emotions. The authors suggest that VR can simulate real-life situations for children to explore safely, and create environments that are difficult to experience in everyday life.

Simeoli et al. [6] propose a new method for diagnosing ASD based on motor abnormalities using a software tool that captures detailed information about children’s motor patterns through a smart tablet device. The study involved comparing the movement trajectories of 30 children with ASD and 30 typically developing children and identified autism with 93% accuracy. The study’s results suggest that this method could provide a new means for assessing young children with ASD and a starting point for rehabilitation treatments. In another example, Regaa et al. [7] examined the effectiveness of a video modeling intervention for social and emotional behavior and skills in children with ASD using a tablet PC. The study showed a marked improvement in emotional skills, highlighting the potential of video modeling as an effective technological tool for intervention and rehabilitation of children with ASD.

Robots have been used in a variety of assistive scenarios, such as meeting various human needs and assisting individuals with ASD to achieve their full potential [8]. The clinical use of social or interactive robots appears to be promising for improving the social skills of children with ASD. Humanoid robot-assisted teaching and intervention programs for children with ASD are rapidly evolving [9].

Socially Assistive Robots (SARs) are a potential strategic technology that could help support interventions for children with ASD, while Robot-Assisted Autism Therapy (RAAT) is an assistive technology application involving robots that are used to support autism therapy [8]. For RAATs to be effectively integrated into real-world treatment for individuals with ASD, they should follow current evidence-based practices used by therapists, such as Applied Behavior Analysis (ABA) [10].

In this study, we propose a system that aims to use robots to assist therapists during therapy sessions with autistic children, particularly in determining the children’s engagement. Furthermore, it is proposed that employing and designing RAAT will assist Arabic-speaking autistic children in becoming more open to social contact by supporting their social development through robot-based interactive therapy. To assess the potential efficacy of this therapy, the children’s engagement during each session was measured and analyzed.

The remainder of this paper is arranged as follows: Section 2 introduces the main concepts of this study with background details; Section 3 presents the system analysis and design; Section 4 describes the materials and methods used to conduct our experiment; Section 5 discusses the evaluation results; and, finally, Section 6 presents the conclusion and future work.

## 2. Background

Four topics are key to this study, namely, Autism Spectrum Disorder (ASD), Applied Behavior Analysis (ABA), Socially Assistive Robotics (SAR), and Robot-Assisted Autism Therapy (RAAT). Each of these will now be discussed in some detail.

### 2.1. Autism Spectrum Disorder (ASD)

ASD is a neurological and developmental disorder that typically begins in early childhood. Individuals with ASD face challenges that may affect how they communicate, interact, behave, and learn. The development of their learning, cognitive, thinking, and social skills can vary considerably from one individual to another. The National Autistic Society (NAS) categorizes them according to impairments in the following three areas:

1. Social interaction; an inability to handle or recognize their own and others’ emotions or understand social cues.
2. Social communication; difficulty in using and understanding verbal and nonverbal language. This also might cause the absence of speech or facial expressions.
3. Imagination; an inability to generalize the skills learned in a particular environment and apply them to different environments or to imagine new situations, which might cause repetitive behavior [11].

Social behavioral therapy (SBT) for ASD focuses on functional independence and quality of life by targeting the development of emotional regulation, social skills, and communication [12]. During SBT sessions, therapists face challenges in working with children with ASD who have difficulties communicating and interacting with others, display repetitive behaviors, and show little interest in social activities. SBT interventions targeting emotional regulation, social skills, and communication are based on Applied Behavior Analysis (ABA), which employs specific teaching methods to develop language, cognitive and sensorimotor skills, social interactions, everyday living skills, and address specific problem behaviors [13].

### 2.2. Applied Behavior Analysis (ABA)

Applied Behavior Analysis (ABA) is a type of therapy that uses principles of learning and motivation to improve social, communication, and learning skills for children with autism [2]. ABA uses a behavioral theory approach that focuses on teaching children to communicate actively and effectively, improve their social development, minimize inappropriate behaviors, develop academic abilities, and enhance their independence in ways that are tailored to each child. Research has shown that intensive and long-term ABA therapy can improve outcomes for most children with autism, including gains in intellectual functioning, language development, daily living skills, and social functioning [14].

### 2.3. Socially Assistive Robotics (SAR)

A robot is a programmable machine capable of carrying out complex actions automatically [15]. Robotics is a rapidly growing field with applications in education, healthcare, environmental work, engineering, and manufacturing, among others [14]. Robots may be pre-programmed, semi-autonomous, or fully autonomous, depending on their degree of autonomy [16].

Socially Assistive Robotics (SAR) is an area within the broader field of Human–Robot Interaction (HRI) that focuses on assisting people in social interactions. SAR aims to develop efficient interactions for therapeutic and educational contexts, including addressing the challenges faced by individuals with autism in social learning, communication, and interaction [14,17].

Four classes of social robots have been identified: socially evocative, social interface, socially receptive, and sociable robots, each with varying levels of capability for social interaction in complex environments. The higher the robot is on the list, the more capable it is of social interaction in complex environments [18]. 

Additionally, Choi et al. [19] found that the design of social robots can affect humans’ emotional engagement with the robots. Autonomous robots were perceived as more intelligent, while tele-operated robots were perceived as having greater social presence. The authors also discussed the implications of these findings for the design of social robots to enhance emotional engagement with humans.

Despite their potential benefits, social robots face significant challenges related to user acceptance, as well as the robustness and degree of autonomy of the robot [20,21]. Srinivasan et al. [22] found that children lost interest in interacting with robots after a certain period, but their study did not tightly control and systematically manipulate individual elements of the robot (such as animate, inanimate, humanoid, and mobile) therapies. User acceptance is often related to the appearance and tactile qualities of the robot used. Researchers have identified design recommendations for Socially Assistive Robots in health and social care, emphasizing the need for involvement from all stakeholders, including robot developers, to produce appropriate social assistive robotics [23,24].

### 2.4. Robot-Assisted Autism Therapy (RAAT)

Robot-Assisted Autism herapy (RAAT) is an assistive technology application involving robots that are used to support autism therapy. It has been used in various fields, such as health and education, to support the developmental needs of autistic individuals, including sensory development, communication, interaction, cognitive development, social development, emotional development, and motor development [23].

RAAT is an emerging field, and there are concerns as to the efficacy of using robots in autism therapy [25]. There are currently only a limited number of studies on the efficacy of RAAT; however, research has shown that using RAAT in treatment sessions can motivate children with ASD to participate in activities.

Different design features and appearances have been suggested and investigated to increase therapeutic efficiency. In studying the potential therapeutic role of robots in autism therapy, some researchers have used humanoid robots, while others have used non-humanoid robots. Giullian et al. [26] proposed a set of requirements that would help engineers design and build robots for use in autism therapy, summarized as follows:

- Appearance requirements; visual appeal, realism, size, and shape must be taken into consideration when designing a robot. For example, a robot with a neutral-colored torso and a face with distinct features is preferable for creating facial cues. The study found that autistic children are more likely to be engaged by a mechanical or mascot-like appearance rather than something that is overly human in appearance.
- Functionality requirements; the senses of reward, locomotion, and choice/control must be taken into consideration when designing a robot.
- Safety requirements; the design of the robot must be safe, as the target user group is children, and this means a robust design, free from sharp edges and exposed wiring.
- Autonomy; the robot must have a level of autonomy, using AI and machine learning methodologies to control its actions and execute them in sequence. The robot does not have to be fully autonomous, as these are not intended to replace the human therapist.

## 3. System Analysis and Design

This section describes our considerations for an RAAT implemented for clinical settings in autism therapy. It presents an overview of the technology involved, the applied context, and the system design approach.

### 3.1. Conceptual Framework

**Figure 1:**
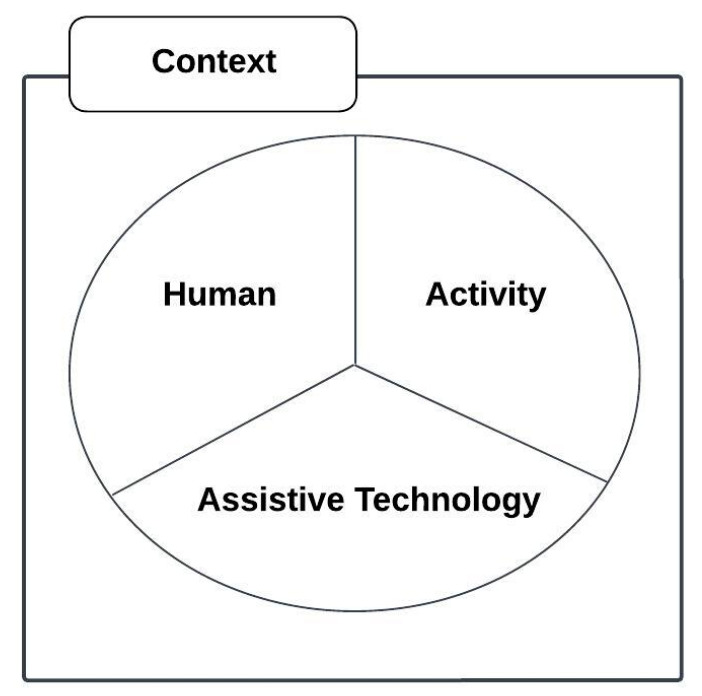
Human, activity, assistive technology (HAAT) model.

The conceptual framework is based on the human, activity, assistive technology (HAAT) framework as proposed by Cook and Hussey [27], modified from a general model for human performance by Yavelberg in [28] and shown in Figure 1.

This is a framework for understanding the place of assistive technologies in the lives of people with disabilities that guides clinical applications and research to enhance a person’s capabilities.

**Figure 2:**
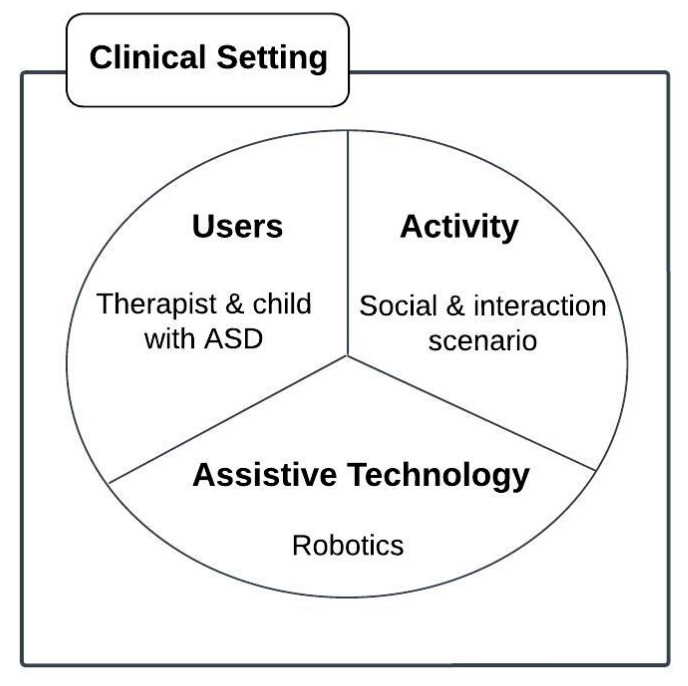
System conceptual framework.

We proposed our conceptual framework to illustrate the main idea behind using robotics as assistive technology to help children with autism in their social interaction activities, as shown in Figure 2.

This framework is proposed to illustrate the variables affecting the use of robotics for children with ASD in a clinical setting to improve their social interactions. The intersection of these variables represents the user, activity, and technology match. The four variables are described below:

1. Users: The users in the framework are those who will benefit from employing the technology and may include parents, family, doctors, nurses, managers, and engineers. In our conceptual framework, the therapist and children with ASD benefit from using robotics in a clinical setting.
2. Activity: The key linkage for using technology is the activity in the conceptual framework which matches the individualized goal(s) for users. In our conceptual framework, we use robotics to support social interaction in the daily activities of children with ASD.
3. Assistive technology: The main principle is the technology used to help the users in the targeted activity and includes various recent innovations that can be deployed to support assistive technologies, such as VR, AI, ML, and robotics. In our conceptual framework, we use robotics in the context of autism therapy in a clinical setting.
4. Context: The selection and use of technology can occur in multiple settings, including the home, the school, and the community. For the purposes of this study, the context is limited to a clinical setting.

### 3.2. The Proposed Framework

Our proposed system aims to use robots to assist therapists during therapy sessions with autistic children, particularly in determining the autistic children’s engagement while they are socially interacting with the robots. This involved creating social interaction scenarios with autistic children and robots to measure their engagement with the robots. These measurements were further validated using assessment forms that were filled out by the observing therapist.

**Figure 3:**
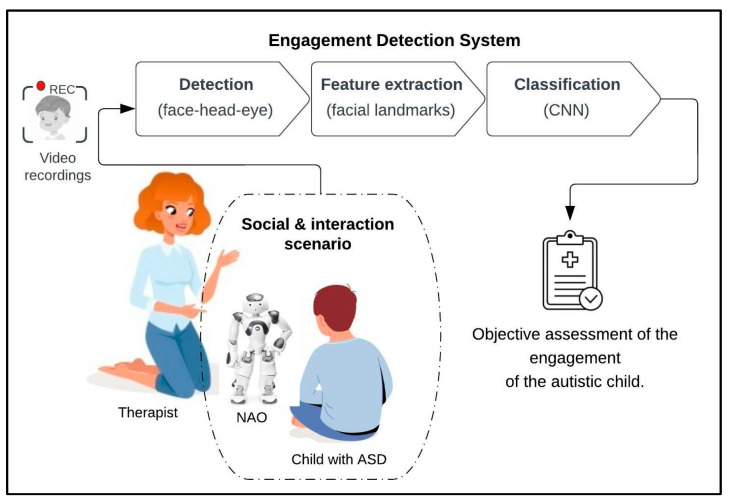
System framework.

Figure 3 illustrates our proposed framework in which the system is designed for use in a clinical setting to measure the engagement of children with ASD during a therapy session. Each autistic child will participate in the social interaction scenarios laid out in the protocol. All the sessions conducted during the experiment are recorded via the robot camera and used in the engagement detection system. Video recordings will go through the processes of face detection, features extraction, and classification to provide an objective assessment of the engagement of autistic children.

We aim to test how a robot’s use of different types of feedback can influence children’s engagement through different scenarios. These feedback types are decided by professionals in autism.

In general, we plan to follow the recommendations given by the researchers in [29], who have previously worked with the Nao robot. As presented in Figure 3, the engagement detection system consists of three steps:

1. Face detection: This step applies machine learning algorithms to detect human faces from video recordings that are taken during therapy sessions. This step includes face, head, and eye detection.
2. Feature extraction: This step applies machine learning algorithms to extract facial landmarks from the face; these landmarks were detected using Dlip, OpenCV libraries, and a convolutional neural network (CNN).
3. Classification: We used deep learning CNN to classify the frames based on extracted features. Each frame was classified as either “engaged” or “not engaged”, as based on the emotion model described next.

A CNN is a type of machine learning algorithm that is commonly used for image recognition tasks. The main idea behind a CNN is that it can automatically learn to detect useful features in images, such as edges, corners, and textures, without the need for manual feature engineering. This is accomplished through the use of a specialized architecture that consists of multiple layers of interconnected neurons, each of which performs a specific task, such as convolution or pooling [30,31]. The advantage of using a CNN is that it can achieve state-of-the-art performance on a wide range of image recognition tasks, such as object detection, face recognition, and medical image analysis, with relatively little manual intervention. This makes it a powerful tool for a wide range of applications.

### 3.3. Emotion Model

We designed and developed an engagement detection system based on the Russell and Pratt (1980) circumplex model of affect [32]. This model describes all affective states as originating from two basic neurophysiological systems within a circumplex that is characterized by two orthogonal dimensions: valence and arousal.

**Figure 4:**
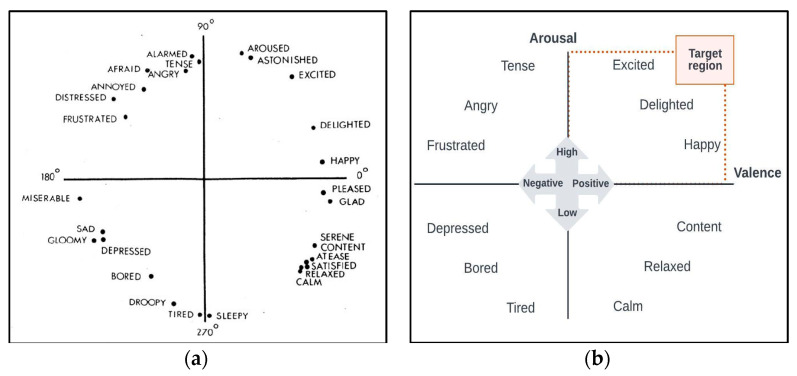
Emotion model: (**a**) Russell’s circumplex model of affect; (**b**) our proposed model of emotion.

Several studies have reported that positive valance and high arousal are indicators of child engagement. In [33], researchers examined the subjective learning experiences and they found that positive valence and high arousal were indicators of emotional engagement. Another research study [24] examined happiness and found that individuals with positive valence and high arousal had higher levels of engagement and satisfaction, and we built our model on this emotion model (see Figure 4).

### 3.4. NAO Robot

**Figure 5:**
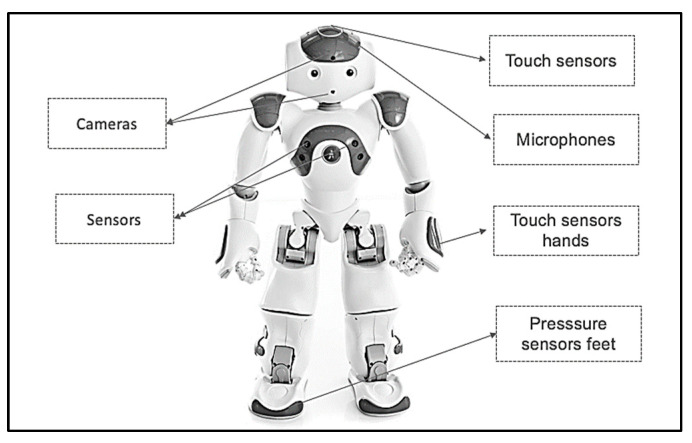
NAO robot.

The robot that is used in this study for robot-assisted therapy is the Softbank Robotics NAO 6, which is a small, human-like robot popular for studies of the child–robot interaction, as shown in Figure 5.

The NAO robot’s programmability and high level of customizability make it a versatile tool for researchers and developers, allowing it to be tailored to a range of applications and scenarios. Additionally, the robot’s interactive and engaging capabilities, such as playing games, storytelling, and dancing, make it an ideal companion for users, particularly in education, healthcare, and research settings. The NAO robot’s key features enable it to perform a wide range of tasks and be utilized in numerous settings [34]. These features include:

- Movement and dexterity: NAO is 58 cm (23 in.) high, with 25 degrees of freedom, can perform a variety of actions with its arms, legs, and head. Its hands are equipped with tactile sensors, enabling it to interact with objects in its environment.
- Vision and hearing: with two cameras and four microphones, the NAO robot can recognize faces and objects, track movements, and respond to sound commands.
- Speech and language: the NAO robot has high-quality speakers and speech recognition software that enable it to communicate in multiple languages and dialects, and understand and respond to voice commands.

These features and capabilities make the NAO robot a promising tool for children and individuals with special needs, promoting social skills and emotional development. It has been widely used in autism therapy [13,24,25,26,27], and its ability to provide a structured and consistent learning environment can enhance engagement levels and support the social development of autistic children.

To implement our proposed scenario on the NAO robot we used Choregraphe software which is a multi-platform desktop application, that allows the creation of animations, behaviors, and dialogs and could test them on a simulated robot, or directly on a real one. We built three scenarios using Choregraphe.

## 4. Materials and Methods

Evaluating the proposed system and scenarios within a clinical context and real-world setting is essential in this study. This provides an opportunity to verify the proposed framework and validate the implemented model. Throughout this section, the experiment design, which includes the procedures used in the experiment and the protocol of the social interaction scenarios between autistic children, is illustrated. Additionally, the participants, data collection, and data analysis are discussed. Insight into the process of collaboration with the Autism Center of Excellence to conduct the experiment is also provided.

### 4.1. Collaboration Process

In the case of earlier studies, an interdisciplinary team of experts identifies the problem at hand together and develops a detailed understanding of it. In our study, developing an appropriate and beneficial robot for autism therapy requires collaboration between researchers, therapists, and medical practitioners.

Therefore, during the research stage, several interviews and focus group sessions were conducted to further understand the problem, collect the requirements, and determine the most suitable protocol for the experiment. In each session, the researcher attempted to ensure that the autism experts and ABA specialists had the opportunity to share their perceptions of the study.

**Figure 6:**
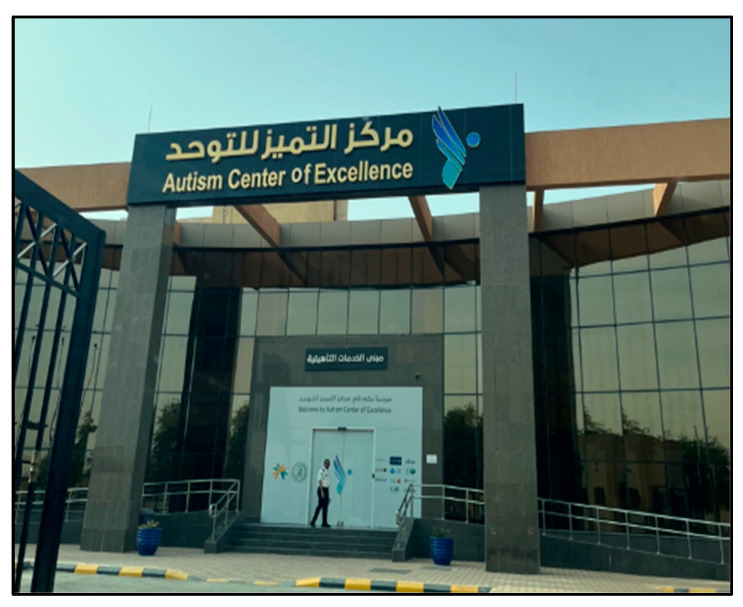
Autism Center of Excellence.

A focus group at the Autism Center of Excellence, Riyadh, Saudi Arabia (Figure 6) was conducted. It involved two ABA specialists, three IT specialists, and a medical practitioner. We focused, in this phase, on identifying the task scenario that is aligned with therapy assessments so that we could incorporate them in the engagement detection system, identifying the case and the sequences of the assisted therapy that are suitable for interaction with the robot.

### 4.2. Ethical Statement

The experimental protocol was reviewed by the Institutional Review Board of the College of Medicine at King Saud University (Ref No. 22/0154/IRB Research Project No. E-21-6377). Informed consent from all participants was obtained prior to enrolling them in the study to ensure that their participation was of their own free will. Each participant was additionally made aware that they had the right to withdraw at any time, and the results of the study would be published at a later date.

### 4.3. Participants

Initially, the prototype was tested on three typically developed (TD) children before being conducted on children with ASD. Our inclusion criteria: a medical diagnosis of ASD confirmed by a clinical psychologist using ADOS-2 [35]. Our exclusion criteria: children with severe ADHD were excluded due to the difficulty of conducting the experiment since they required more sessions to familiarize them with the robot which was difficult due to experiment time constraints, since the experiment was conducted just before the summer vacation. 

We recruited 5 participants, their ages ranging between 5 and 10 years old. They had moderate to severe ASD which is indicated in the table as 2 or 3 for ASD level (see Table 1).

It should be noted that none of the children had vision problems at the time of the study, except for child number four, who was wearing glasses. The study population is native Arabic-speaking autistic children within the age group of 4–11 years old.

### 4.4. Protocol

Children with ASD take great interest in socially interacting and communicating with robots. Additionally, many studies suggest that children with ASD desire robots as trainers to improve their social interaction and communication abilities, which are often impaired due to their disorder [25]. Therefore, the research presents social interaction scenarios with autistic children in this section.

Prepared in cooperation with an ABA specialist, the proposed protocol included social interaction scenarios that ran throughout a twenty-minute therapy session for each autistic child. A humanoid NAO robot was employed during the therapy sessions, and participant videos were captured to analyze facial expression activities to measure engagement. An outline of the protocol can be reviewed in Table 2 below.

**Figure 7:**
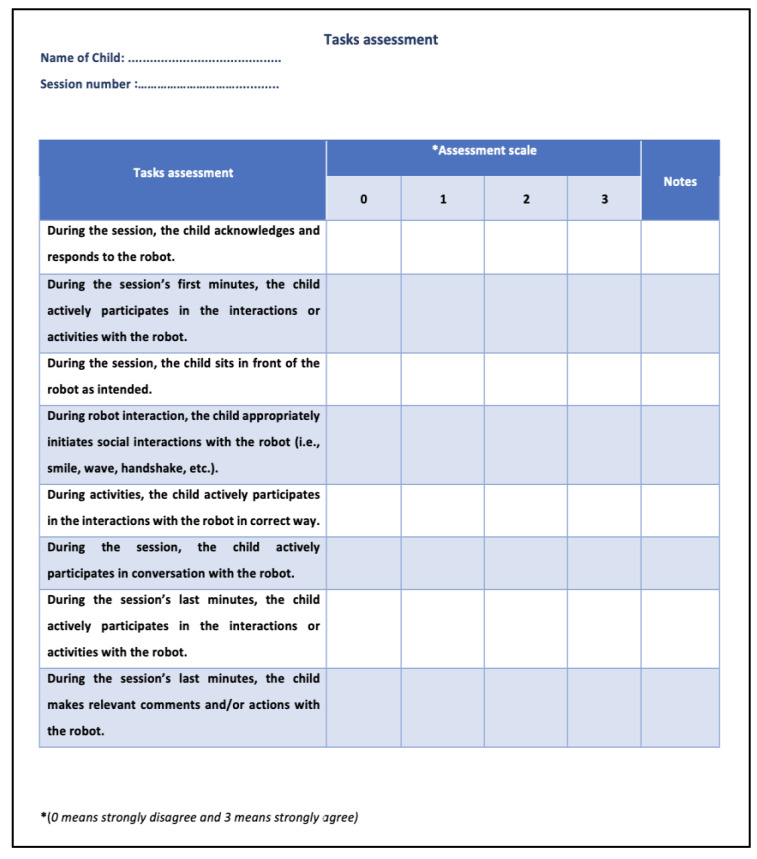
Behavior-based assessment sheet.

During the sessions with the robot, the children’s behavior was evaluated. Assessment Figure 7 was used to validate the proposed solution, which was designed with the therapist. It outlines the task evaluation and contains the task assessment and assessment scales (0 to 3). On the scale, “0” means strongly disagree whilst “3” means strongly agree. For each task, the maximum score of this assessment was 24 points.

### 4.5. Experiment Setting

**Figure 8:**
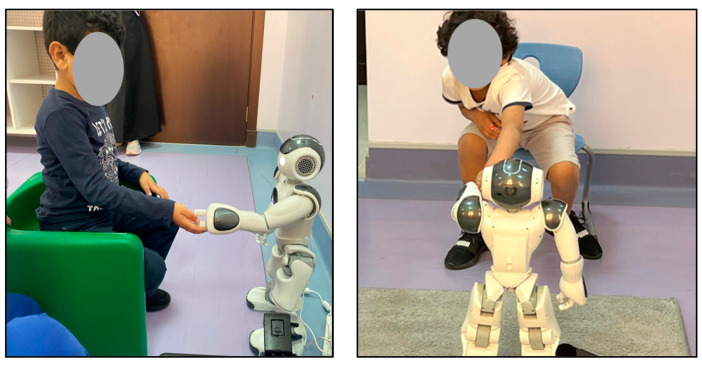
Two recording sessions.

The setting of this experiment was a quiet room in the Autism Center of Excellence, Figure 8 includes some images that were captured during the experiment. As can be seen, the robot was placed at a distance of at least one meter from the child. During the experiment and interaction with the robot, the child could select whether to sit on a chair or on the floor.

### 4.6. Test Protocol with TD Children

**Figure 9:**
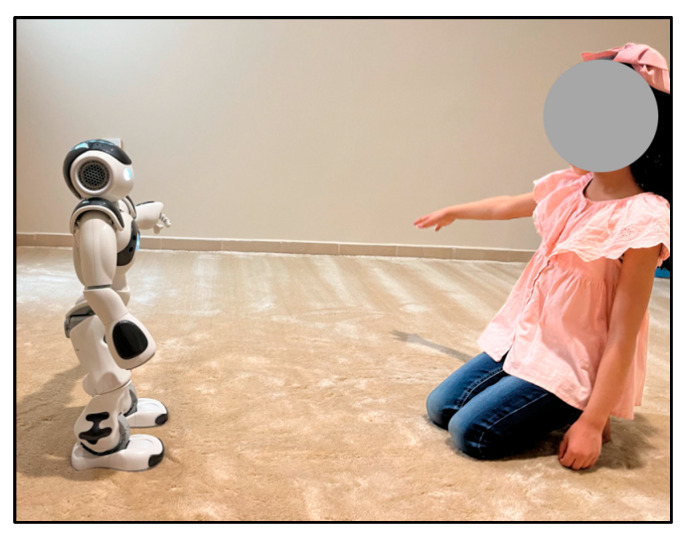
Test with TD children.

To ensure the safety of the NAO robot with autistic children, the proposed experiment was tested on three TD children (see Figure 9) with a sample age group of 4–11 prior to experimenting with autistic children. Testing with (TD) proceeded as follows:

- Encourage the TD child to interact with the robot casually.
- Observe the usability of the robot and how the child interacts with it.
- Test the social interaction scenarios that were planned.
- Review the safety of using this type of robot with the children.
- Modify the social interaction scenarios if need be.

In addition, the video recordings captured were analyzed using the proposed engagement detection model. By reviewing the videos, it can be seen that all TD children had a good reaction as they were happy and excited to be interacting with the NAO robot.

### 4.7. Data Collection

**Figure 10:**
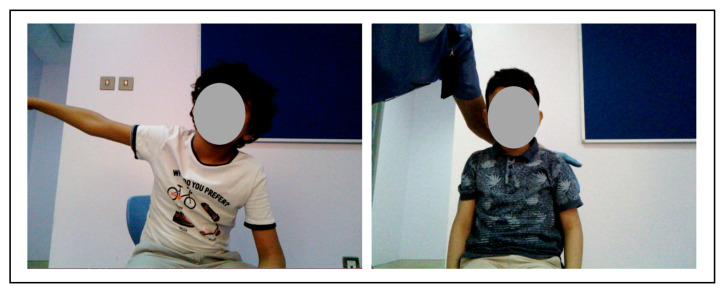
Recorded frame.

All sessions conducted during the experiment were recorded via the NAO robot camera and later used for analysis to detect engagement. Video recordings went through the processes of face detection, feature extraction, and face classification to examine autistic children’s engagement. Throughout the experiment, it was paramount to ensure that the child’s whole face was seen clearly by the robot’s camera to guarantee that the algorithm could detect all facial expressions (Figure 10 shows a suitable example of detected frames). Three recordings were collected for each child.

### 4.8. Data Analysis

Initially, pre-processing is necessary since the datasets are in video format, which needs to be converted into images, which are called frames. We pre-processed the input video files in order to extract the desired frames. OpenCV (Open Source Computer Vision) is an open-source library that includes several hundred computer vision algorithms and is widely used in computer vision generally, and facial recognition more specifically [36,37]. Using the OpenCV library, we were able to read the video streams before creating a VideoCapture object and splitting them into frames. The Dlib library was used for detecting and cutting out the faces in each frame.

Features extraction is an important step in the facial recognition process. Face landmarking assists in identifying and representing salient areas of the face, including the eyes, eyebrows, nose, mouth, and jawline. OpenCV, Imotion, MTCNN, and Open Face are among the open-access software packages available for the automatic and efficient detection of facial landmarks. In our implementation, we used the Dlib library and OpenCV which can detect and extract facial landmarks from each frame. With the help of Dlib, a facial landmark detector that has pre-trained models, 68 coordinates were estimated on a person’s face (x, y) [37].

We created a Python script that read all the frames, and for each frame, we detected the face and extracted the 68 coordinates (x, y) which were then saved in a text file and then saved into a data frame. For feature classification, we applied two convolution layers with 32 filters and (3, 3) kernel size. Then, we applied the third convolution layer which consists of 64 filters and (3, 3) kernel size. After each convolution layer, there is an activation function called ReLU to set all the negative pixels to 0. This function introduces non-linearity to the network and generates an output-rectified feature map [38,39,40]. After the ReLU operation, there is a pooling layer for simple and salient elements. Finally, the flattened layer produces the output class (engagement/non-engagement).

## 5. Results and Discussion

In this section, we present the result of the experiment by presenting the results of each child using the proposed engagement detection system using the CNN model, and the manual assessment from the therapists in the Autism Center of Excellence.

To calculate the engagement of each child, a script using Python code was implemented to read each video. Furthermore, each video was split into frames before being processed within the model to determine whether the child was engaged. This was completed by calculating the percentage of the engagement as per Equation (1) [41]. The engagement percentage calculated using the CNN model as EPM was then denoted.

(1) $$\mathrm{EPM}=\frac{Nb\;of\;frames\;that\;are\;classified\;as\;engaged\;}{total\;number\;of\;frames}\times 100\;$$

To quantify the engagement of each child based on the therapist’s assessment, the assessment sheet contains a scale ranging from 0 to 3. In this study, “0” means strongly disagree whilst “3” means strongly agree. The findings were then converted to a percentage using Equation (2) [41]. We then denote the engagement percentage of the therapist as EPT.

(2) $$\mathrm{EPT}=\frac{Sum\;of\;total\;points}{Mximum\;possible\;pointser\;of\;frames\;}\times 100$$

Table 3 details the results, highlighting that percentages calculated from the model versus the therapist assessment were almost identical regarding children one and three. However, the results for children two, four, and five were below 50% for both assessments. The most significant difference in the assessment was noted in child five. Although it is believed that engagement detection of the model may have been affected as child four was wearing glasses, child five had a severe level of autism. Child five’s reactions were not evident, but according to the treating therapist, the child’s reactions were considerable given the baseline reactions. Therefore, the discrepancy in results concerning the proposed model can be seen.

The Cohen kappa test [42] was applied to evaluate the agreement level between the therapist assessment and the proposed model result. The Cohen kappa test is a statistical measure of inter-rater reliability used to determine the level of agreement between two or more raters or observers. It takes into account the possibility of chance agreement and provides a more accurate assessment of the level of agreement than simple percentages of agreement. The kappa coefficient ranges from −1 to 1. It is interpreted as follows: values ≤ 0 indicate no agreement, 0.01–0.20 indicate slight, 0.21–0.40 indicate fair, 0.41–0.60 indicate moderate, 0.61–0.80 indicate substantial, and 0.81–1.00 indicate an almost perfect agreement. Cohen’s kappa looked at an agreement as a nominal variable. We considered 50% and above as an engaged label and below 50% as a non-engaged label. The result score indicates a perfect agreement level.

Considering the above, it can be suggested that the proposed system offers some accuracy in measuring the engagement of children with ASD. It is also believed that the system provides results that could support therapists during the assessment of ASD children upon interacting with the robot. Meanwhile, it can be said that robot-assisted therapy is a promising field of application for intelligent social robots, especially when supporting children with ASD.

During the final stage of the experiment, we obtained expert reviews from four autism therapists. The feedback was positive, with some professionals stating, “We have not seen the kid so excited like that”, and ‘‘The robot attracted the attention of the child greatly”. However, additional reviews indicate that the professionals are unsure whether the robot offers long-term social change. Although this is true, professionals within the field have indicated that robot incorporation is promising. Recommendations, such as considering different ages, different autism severity levels, and different therapy stages for testing the robot with different case studies, were suggested.

Autism therapists also identified areas where the robot’s incorporation into therapy sessions could be enhanced. They identified several important factors that influence robot adoption and effectiveness, including cost, safety, and utility. These key factors are crucial in gaining acceptance of the robot as a clinical assistive tool. The cost of the device, the time it takes to set it up, and the time it takes to train are all cost factors. Side effects and safety certification are two additional important considerations, as is system usability.

## 6. Conclusions and Future Work

Our proposed system aims to use robots to assist therapists during therapy sessions with autistic children, particularly in determining the children’s initial engagement. Furthermore, we propose that employing and designing RAAT will assist Arabic-speaking autistic children in their social development through robot-based interactive therapy. To measure the children’s engagement during each session, we used observational methods and analyzed the data collected.

While the results of our study suggest that the robot-based intervention may have potential for supporting children with ASD in social engagement, we acknowledge that the current research design has limitations, such as the small sample size, brief interaction duration, and lack of a control group. Therefore, further research is needed to confirm and extend these findings. Future studies could investigate the long-term effects of the intervention and compare the effectiveness of the robot-based intervention to other interventions. Additionally, the use of larger, more diverse samples could provide a more comprehensive understanding of the potential benefits and limitations of robot-based interventions for individuals with ASD. Furthermore, social interaction ability of the robot, engagement level, length of sessions, and child engagement should all be assessed and measured within one framework to provide comprehensive results.

Overall, robot-assisted therapy is a promising field of application for intelligent social robots, especially to support children with ASD in achieving their therapeutic and educational objectives, i.e., social and emotional development, communication and interaction development, cognitive development, motor development, and sensory development. We anticipate that the challenges faced will be addressed soon with expert interdisciplinary collaboration.

Future directions for research include expanding the sample size and conducting several clinical trials to assess the potential effectiveness of our proposed system to assist autism therapy. Design considerations can be extended to produce online and real-time analysis and visualization, and the experimental protocol can be integrated to become a part of the autism therapy session to support the autistic specialists. In addition, we aim to increase the performance and robustness of the system. We may also consider other modalities, such as detecting the emotions of the children using speech and skin conductivity.

## Figures and Tables

**Table 1:** Participants.

Children	Age	ASD Level	Gender
C1-ASD	8	2	Male
C2-ASD	8	2	Female
C3-ASD	5	2	Male
C4-ASD	10	2	Male
C5-ASD	7	3	Male

**Table 2:** Experiment protocol.

#	Scenario		Description
1	Identify the robot.		The child is instructed on how to interact and communicate with the NAO robot.
2	Familiarize the child with the robot.		The robot says, “Salaam alaikum”.
			This activity aims to build a friendly relationship between the child and the robot at the beginning of the experiment.
3	Start the first phase of the experiment.		Once the child feels comfortable with the robot, the main tasks begin. First, in the training session, the robot is placed at a distance of at least one meter.
4	Task#1	Call the child’s name.	The robot says, “Salaam alaikum” and calls the child’s name.
5	Task#2	Greeting.	The robot tries to shake the child’s hand.
6	Task#3	Imitation exercise.	The robot raises its right hand, and the child tries to imitate it.

**Table 3:** Result analysis.

Child	Scenarios	Time (s)	# Frames	CNN_ASD		Therapists Assessment
				% Engagement	% Rate	
C1-ASD	Task 1	19	186	65%	84%	88%
	Task 2	8	84	88%		
	Task 3	19	187	98%		
C2-ASD	Task 1	10	95	2%	14%	29%
	Task 2	11	108	14%		
	Task 3	20	199	24%		
C3-ASD	Task 1	12	124	52%	41%	42%
	Task 2	11	114	0%		
	Task 3	2	25	72%		
C4-ASD	Task 1	5	45	7%	15%	21%
	Task 2	5	45	22%		
	Task 3	5	52	16%		
C5-ASD	Task 1	19	187	0%	9%	42%
	Task 2	11	107	28%		
	Task 3	19	187	0%		

## Data Availability

Not applicable.
